# Policy review on the management of pre-eclampsia and eclampsia by community health workers in Mozambique

**DOI:** 10.1186/s12960-019-0353-9

**Published:** 2019-02-28

**Authors:** Salésio Macuácua, Raquel Catalão, Sumedha Sharma, Anifa Valá, Marianne Vidler, Eusébio Macete, Mohsin Sidat, Khátia Munguambe, Peter von Dadelszen, Esperança Sevene, the CLIP Working Group, Jeffrey Bone, Alison Maclean, Maggie Woo Kinshella, Tang Lee, Jing Li, Beth A. Payne, Kien NhanTu, Sharla Drebit, Asif Raza, Dustin Dunsmuir, Ana Ilda Biza, Dulce Mulungo, Ernesto Maximiano, Silvestre Cutana, Charfudin Sacoor, Helena Boene, Felizarda Amosse, Paulo Filimone, Corssino Tchavana

**Affiliations:** 1 Centro de Investigação em Saúde de Manhiça (CISM), Manhiça, Mozambique; 2 Department of Obstetrics and Gynaecology, University of British Columbia (UBC), Vancouver, British Columbia Canada; 3 Ministério de Saúde, Maputo, Mozambique; 4 Universidade Eduardo Mondlane, Faculdade de Medicina, Maputo, Mozambique; 5 School of Life Course Sciences, Faculty of Life Sciences and Medicine, King’s College London, London, United Kingdom

**Keywords:** CHWs, Health policy, Maternal health, Pre-eclampsia and eclampsia, Mozambique

## Abstract

**Background:**

Pre-eclampsia is one of the leading causes of maternal death in Mozambique. Limited access to health care facilities and a lack of skilled health professionals contribute to the high maternal morbidity and mortality rates in Mozambique and indicate a need for community-level interventions. The aim of this review was to identify and characterise health policies related to the role of CHWs in the management of pre-eclampsia and eclampsia in Mozambique.

**Methods:**

The policy review was based on three methods: a desk review of relevant documents from the Mozambique Ministry of Health (*n* = 7), contact with 28 key informants in the field of health policy in Mozambique (*n* = 5) and literature review (*n* = 699). Policy documents obtained included peer-reviewed articles, government and institutional policies, reports and action plans.

Seven hundred and eleven full-text documents were assessed for eligibility and included based on pre-defined criteria. Qualitative analysis was done to identify main themes using content analysis.

**Results:**

A total of 56 papers informed the timeline of key events. Three main themes were identified from the qualitative review: establishment of the community health worker programme and early challenges, revitalization of the CHW programme and the integration of maternal health in the community health tasks.

In 1978, following the Alma Alta Declaration, the Mozambique government brought in legislation establishing primary health care and the CHW programme. Between the late 1980s and early 1990s, this programme was scaled down due to several factors including a prolonged civil war; however, the decision to revitalise the programme was made in 1995.

In 2010, a revitalised programme was re-launched and expanded to include the management of common childhood illnesses, detection of warning signs of pregnancy complications, referrals for maternal health and basic health promotion. To date, their role has not included management of emergency conditions of pregnancy including pre-eclampsia and eclampsia.

**Conclusion:**

The role of CHWs has evolved over the last 40 years to include care of childhood diseases and basic maternal health counselling.

Studies to assess the impact of CHWs in providing services to reduce maternal morbidity and mortality are recommended.

## Background

In 2015, it was estimated that 99% (302 000) of the global maternal deaths occur in developing regions being 66% (201 000) in sub-Saharan Africa [1]. They mostly affect vulnerable populations with poor socioeconomic background from remote areas with limited access to health care services [2]. Although Mozambique witnessed a 65% decrease in maternal mortality between 1990 and 2015 (1390 to 489 maternal deaths per 100 000 live births), this failed to meet the target set by Millennium Development Goal (MDG) 5A in 1990 to reduce maternal mortality by two thirds by 2015 [1].

Despite the difficulties in obtaining reliable data on estimates of maternal mortality and its causes [3], hypertensive disorders of pregnancy are the third major cause of maternal death [4–6] and the second major cause of near misses in Mozambique [7].

It is estimated that pre-eclampsia, a systemic syndrome characterised by new-onset hypertension and proteinuria in pregnancy, results in 25 000 maternal deaths in Africa annually, accounting for around 9% of all deaths [8]. Severe pre-eclampsia is also associated with significant maternal morbidity (e.g. stroke and liver rupture) and adverse perinatal outcomes such as prematurity [9]. Pre-eclampsia is a particular problem due to the nature of the condition and the need for early identification and management to prevent complications [10]. Although there is no universally accepted standard of care for the condition, which is dependent on the locally available resources and facilities, it is widely agreed that risk reduction for women with pre-eclampsia includes standardised assessment and surveillance, management of severe hypertension and prevention and management of the seizures of eclampsia [10].

In many low- and middle-income countries (LMIC), access to health care facilities and skilled health professionals able to respond to obstetric complications, including pre-eclampsia and eclampsia, is limited [11]. In Mozambique, only 50% of the population has access to primary health care, and just 36% live within 30 min of a health facility [12]. Antenatal care and delivery services are free of charge in Mozambican public health facilities; however, lack of access to transport and life-saving drugs present strong barriers for care seeking [13]. The unavailability of trained and qualified health workers is a significant problem. Although the number of health workers is increasing, it fails to reach the growing needs of the population. In 2000, there were 2.5 doctors and 21.25 nurses per 100 000 people in Mozambique, significantly less than the African average of 21.7 doctors and 117 nurses per 100 000 people [14]. These barriers to healthcare access influenced the decision to revitalise the country’s community health worker (CHW) programme [15]. Despite several challenges, Mozambique has a longstanding programme of CHWs, called *Agentes Polivalentes Elementares* (APEs) [16], who are lay people selected by the community where they live to serve as a link between the community and the primary health care system. They do not have a formal or professional education. However, they are trained to deliver health promotion and disease prevention advice in hard-to-reach rural populations, under the supervision of local health care providers [16, 17].

CHWs have been found to have a role in the reduction of maternal mortality in other settings; therefore, CHWs may also be instrumental in improving maternal health in Mozambique [18].

The aim of this review was to describe policies related to the role of CHWs in the management of pre-eclampsia and eclampsia in Mozambique since the creation of the programme.

## Methods

### Study area

Mozambique is a low-income country with around 60% of the population living below the poverty line of 1.25 dollars a day [19]. Most of the population (62% in 2014) lives in rural areas, and a large proportion has no access to health facilities [20]. Almost two thirds (62%) of women aged 15–49 report problems in accessing health services; distance to health facilities was the most commonly cited barrier [21]. In the 5 years between 2007 and 2011, the proportion of births attended by a skilled health professional was 54% [21, 22].

### Study design

This descriptive study was based on a formative research exercise conducted in preparation for the Community Level Interventions for Pre-eclampsia (CLIP) Trial, a multi-country study that aims to reduce the burden of adverse maternal and perinatal outcomes related to pregnancy hypertension through community engagement and mobile health-supported task-sharing to community health care providers (NCT01911494).

The policy review was based on three methods: (1) a desk review of relevant documents from Mozambique’s Ministry of Health (MoH), (2) key informants consultations and (3) a literature review.

#### Desk review

For the desk review, formal government and institutional policies and other relevant official documents, such as community involvement strategies, community health worker training programmes, monitoring and evaluation manuals, feasibility study reports and meeting’s minutes were collected from Mozambique’s MoH. Some of these official documents were accessed through the Mozambican Government Portal; others were only available in hardcopy within the MoH. To access hardcopy documents, the researchers visited the MoH office in Maputo, where relevant documents were shared for review and notes were taken regarding key findings related to CHWs in Mozambique. The search of the government portal was conducted using the following keywords: CHWs, CHW training, CHW curriculum, CHW programme, health policy, community policy, maternal health, eclampsia and pre-eclampsia. This search was limited to documents published in English or Portuguese from 1970 to October 2017.

The documents were reviewed by SM and RC to determine the timeline of policy development and key events.

#### Key informants

A total of 28 key informants (Table 1) were approached for further information regarding the documents found in the desk review. The informants were heads of relevant programmes and organisations, or equivalent, such as CHW programme at central and at the provincial level, maternal health programme, community engagement programme, *Associação Moçambicana de Obstetras e Ginecologistas* (AMOG), NGOs, community leaders and Mozambican training institutions with insiders’ knowledge of health policy development in the country. SM and ES approached the key informants at their working sectors to conduct about an hour long face-to-face informal conversations; field notes of relevant information were taken. Discussions focused on the policy strategies and guidelines, events and timeline, CHWs role and its evolution in maternal health.

**Table 1:** Key informants

Key informants	Number of key informants
CHW programmes in MoH, Maputo and Gaza province	5
Maternal health programme in MoH	2
Pharmaceutical Department in MoH	1
*Associação Moçambicana de Obstetras e Ginecologistas* (AMOG)	2
Mozambican training institutions	4
NGOs	2
Community leaders	12
Total	28

#### Literature review

An electronic search was conducted in two databases: PubMed and HINARI. Keywords related to the study subject were combined with MeSH terms for the search: ‘Mozambique’ and ‘community health workers’, ‘community practices’, ‘evidence-informed policy’, ‘community policies’, ‘health policies’, ‘health systems’, ‘pregnancy’, ‘maternal health’, ‘pre-eclampsia’, ‘eclampsia’ and ‘hypertensive disorders of pregnancy’.

The following limits were used: studies published between 1970 and October 2017, in either Portuguese or English language, based on both quantitative and qualitative research methodologies. The electronic search was independently performed by the first author (SM) and then replicated by the co-author (RC).

After the above-described literature search, all abstracts were extracted and screened by both SM and RC. The relevant papers as well as the documents obtained via key informants and desk review were included in this study (PRISMA diagram) using criteria in Table 2.

**Table 2:** Criteria for inclusion of published papers and documents in the study

- Related to community health workers in Mozambique	
- Post 1975	
- Published in either Portuguese or English language	
- Include information about maternal health	
- Be peer-reviewed or published by a reputable government agency or NGO	
- Full text available	

A qualitative content analysis was performed of all documents and articles that met the inclusion criteria. A manifest analysis approach was used. RC and SM independently familiarised themselves with included documents and then manually coded these for meaning units. RC, SM and ES met and agreed on the inductively derived meaning units, making sure all aspects of content had been covered in relation to the study aim. Themes were then derived from the data.

## Results

**Fig. 1:**
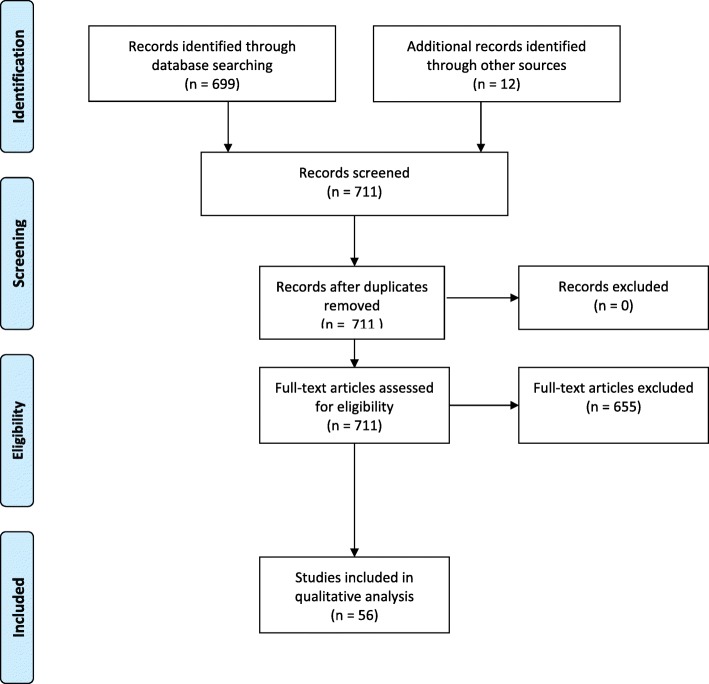
PRISMA diagram

Using the above-described combination of methods, a total of 699 papers were identified from the literature search and a total of 12 papers were obtained via the other two methods of data collection (see Fig. 1). Seven of the documents were obtained from desk review, and five of the documents were retrieved from key informants (Table 3).

**Table 3:** Key documents and corresponding events in the development and evolution of the community health worker programme in Mozambique (1977–October 2017)

Year	Title	Issuing authority	Source of document	Description
1977	‘Formação de Agentes Polivalentes Elementares, II Cadernos de Saúde, II Serie, Número 1’	Ministry of Health (MoH) of Mozambique	Key informant	CHW training manual describing the training package of the first APE programme with focus on health promotion activities
1978	Portaria 46/75 6 de Setembro	MoH	Desk review	Governmental legislation establishing primary health care
1984	The Evolution of Health Policy in Mozambique: Towards a People’s health service.	Walt G, Melamed A, editors, Zed Books Ltd.	Desk review	A peer review article assessing the evolution of health policies in Mozambique post-independence mentioning community engagement
2004	Estratégia de Envolvimento Comunitário	MoH	Desk review	Community engagement strategy for health description of community engagement activities and role of CHWs in the treatment of diseases
2007–2012	Plano Estratégico do Sector da Saúde (PESS)	MoH	Desk review	Strategic plan for health included a new integrated package of services to improve maternal and child health
2009	Plano Integrado para o Alcance dos Objectivos 4 e 5 de Desenvolvimento do Milênio 2009–2012	MoH	Desk review	Strategic plan to reach Millennium Development Goals 4 and 5 (maternal and child health goals) details the role of APE in promoting maternal health
2010	Programa de Revitalização de Agentes Polivalentes Elementares	MoH	Desk review	CHW training programme: detailed description of the new CHW programme, its new scope of work and training
2011	Conteúdo do novo Kit de Agente Polivalente Elementar (APE)	MoH	Key informant	CHW training programme: list of contents of the kit provided to CHWs in the revitalised programme
2011	Manual de Formação de Agentes Polivalentes Elementares (APEs): Manual do Participante	MoH	Key informant	New manual for training of CHWs regarding the CHW revitalised programme
2011	Avaliação e Testagem dos Materiais de Formação dos APEs. Relatório Final	MoH	Key informant	Final report of the assessment of the new training materials for CHWs
2012	Estudo de Base Para Availação do Impacto do Programa de Revitalização dos Agentes Polivalentes Elementares (APEs) na Saúde Comunitária em Moçambique	MoH	Key informant	Background study to assess the impact of the CHW revitalisation programme in the community health of Mozambique
2014	Relatório Anual das Actividades do Programa de Agentes Polivalentes Elementares (APEs) do Ano 2013	MoH	Desk review	Annual report on the CHW programme year 2013

**Fig. 2:**
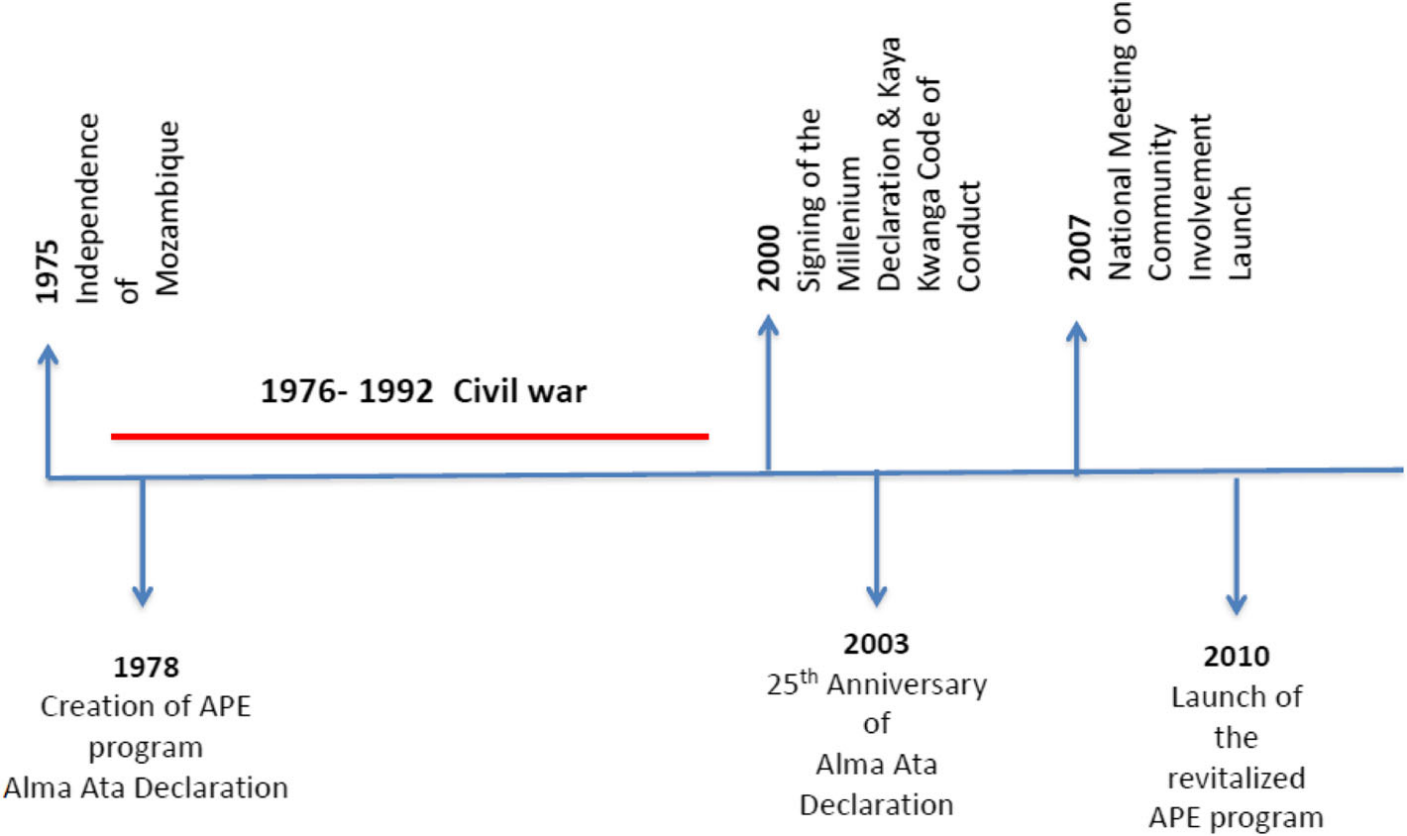
Timeline of relevant events to CHW programme in Mozambique

After assessing the papers using the eligibility criteria outlined in Table 2, a total of 56 papers were included (Fig. 1) which allowed identification of the timeline of key events in the development of the community health worker programme in Mozambique and evolution of CHW’s role in maternal health. A diagram illustrating the key events in the history of Mozambique and development of the CHW programme can be found in Fig. 2.

In addition, three main themes were identified from our qualitative content analysis: the foundation of the CHW programme and early challenges, revitalization of the CHW programme, and CHWs and maternal health, each of which is described below.

### Foundation of the CHW programme and early challenges (1975–2000)

In 1975, when Mozambique gained independence, the government introduced new policies at the time of creation of the National Health Service (NHS) to benefit all Mozambicans [23]. The new policies aimed to strengthen and extend primary health care to rural communities, where there were a disproportionately low number of health workers [17]. This led to the implementation of the CHW programme in 1978 [24]. In Mozambique, CHWs are known as *Agentes Polivalentes Elementares* (APEs), meaning ‘essential multi-purpose agents’; their initial focus was to provide primary healthcare services to remote rural communities [25–27]. CHWs were selected by the health authorities and trained to carry out health promotion and disease prevention activities for the general population without specific focus to maternal health [25].

A 16-year civil war following independence (1976–1992) dramatically damaged the health system as hundreds of health posts were destroyed, and many health workers were unable to perform their duties [28]. The civil war also negatively impacted the CHW programme as it hindered outreach capacity, appropriate supervision and technical support of CHWs [29, 30].

CHWs also worked based on an outdated curriculum (last updated in 1977) with no opportunities for professional development [23, 29]. The number of health workers decreased due to lack of funding; however, several CHWs continued working supported by non-governmental organisations (NGO) [16]. As a result of this system collapse, the CHWs shifted their focus from the preventive tasks that offered little or no financial return towards curative activities they could charge for, but none of them were pregnancy related. [23, 29].

In the late 1980s and early 1990s, increasing numbers of foreign aid agencies and NGOs directed their efforts towards Mozambique in order to tackle the humanitarian crisis created by the civil war. During this period, CHW training implemented by NGOs resulted in multiple ‘vertical programmes’ focussing on singular interventions such as those targeting HIV/AIDS or tuberculosis [29, 31]. NGOs implemented a system of subsidies and provided additional incentives for CHWs involved in these programmes, which led to frustration among volunteer CHWs [32]. In addition to the division of the responsibilities and the above-mentioned compensation of the CHWs, both government-trained and NGO-incentivised CHWs lacked supervision from the MoH [23, 29]. Despite that some of the interventions implemented by NGOs involved in somehow maternal health aspects that were not the priority of these organisations.

It has been argued that the implementation of vertical programmes has fragmented the health system, undermining the local control of health programmes, and has contributed to the reduction of opportunities to introduce new components to the maternal health services [31].

Acknowledging some of these problems, several donors signed the ‘Kaya Kwanga Code of Conduct’ in May 2000 which pledged to ensure that technical assistance is driven by MoH priorities and the avoidance of departure of qualified personnel through contracting of civil servants for donor consultancies as well as departure of CHWs to join vertical programmes run by NGOs offering higher salaries [33].

### Revitalization of the CHW programme (2007–October 2017)

The government made sporadic attempts to revive the CHW programme, for instance through the revision of training and support manuals produced in 1992 and 1993. The MoH continued to supply CHWs with essential medications through a kit system adopted in the 1980s that was updated to reflect the World Health Organization (WHO) essential medicines list [16]. However, these attempts were not successful in reviving the programme due to several sustainability-related factors including the exodus of CHWs to work for NGOs [23].

The 25th anniversary of the Alma Ata Declaration [34] in 2003, which endorsed primary health care as means to provide universal access to health, renewed international efforts for community-based health services and promoted meetings on community involvement at the international and national level [35, 36]. In 2007, the ‘National Meeting on Community Involvement’ took place, where a national strategy for the involvement of the community in health was presented [37].

The national strategy for community involvement in health included the re-launch of the CHW programme (Fig. 2), recognising the important role CHWs play in health promotion and disease prevention in the community [12, 37]. The government aimed to expand health services to 20% of the uncovered population [23]. As of December 2013, there were 2270 CHWs trained across 117 districts, covering 12% of the total population [38].

The revitalised programme required CHWs to visit people in their homes, rather than work from health posts as they had done previously. Each CHW is currently expected to serve a population of 500 to 2000 inhabitants, and their activities can cover a distance of 5–8 km from the referral health facility [23]. In the new model, they belong to the community they serve, are selected by community leaders and endorsed by community members. CHWs must be over 18 years of age, be able to speak the local language fluently and read and perform simple arithmetic; though they can be of any gender, preference is given to women [23].

Acknowledging the role of poor supervision in the failure of the previously established CHW programmes, the 2010 programme established protocols for supervision involving interaction between the province and district supervisors, district and health facility supervisors and health facility supervisors and CHWs [39]. Furthermore, the CHWs are now part of the MoH and receive monthly subsidies [23]. However, the subsidy is minimal, equivalent to 21 US dollars [23], lower than the national minimum wage of 55 US dollars [40].

### CHWs and maternal health care

In 2000, Mozambique signed the Millennium Declaration committing itself to work towards the MDGs, a common development framework shared by the 189 participating countries. Mozambique integrated these objectives in its ‘National Agenda for the Fight Against Poverty’, the ‘2010 Gender, Environmental and Climate Change Strategy and Action Plan’ and in presidential initiatives such as the ‘2008 Presidential Initiative on Maternal and Child Health’ [41]. The 2007–2012 National Health Strategic Plan [Plano Estratégico do Sector da Saúde (PESS), (Table 3)] included a new integrated package of services to improve maternal and child health.

As a result of this strategic plan, the focus is now on health promotion and disease prevention, with official guidelines indicating that 80% of CHW’s time should be spent on these activities and only 20% on curative services [23]. Their 4-month residential training reflects the emphasis on preventive medicine, being curative care only centred on child health [42].

CHWs are provided with equipment to aid in the delivery of services including malaria rapid diagnostic tests, oral rehydration solution for diarrhoea, and antimicrobials for acute respiratory infections and malaria treatment. These medications are part of the Integrated Community Case Management (iCCM) of childhood illness programme embedded in their curriculum and financed mainly by international organisations and NGOs. It has been argued that strong support from these partners led to actions to strengthen iCCM of childhood diseases which culminated with the launching of the CHW revitalization programme [16]. No similar programme for the management of specific maternal health conditions was integrated neither in the CHW’s package of care nor in their training curriculum. In fact, CHW’s curriculum is limited to health promotion in pregnancy. CHWs are trained to encourage pregnant women to attend antenatal clinics, to receive screening (for HIV and other diseases), vaccinations (for tetanus toxoid), and for assessments of foetal size and heart rate. They also help women plan for delivery by encouraging them to organise transport and save money in case of an obstetric emergency. CHWs are also taught to recognise danger signs in pregnancy such as vaginal bleeding and seizures and to facilitate a safe transfer to a local health facility if appropriate [43].

Accordingly, the Integrated Plan for the Achievement of the MDG 4 and 5 [Plano Integrado para o Alcance dos Objectivos 4 e 5 de Desenvolvimento do Milénio 2009–2012 (Table 3)], developed by the Mozambique’s MoH in collaboration with WHO and other international organisations, incorporated CHWs in the minimum care package required to be delivered to achieve these objectives, and singled out family planning and prevention and screening of HIV and other sexually transmitted infections as CHWs main duties regarding maternal health. No curative or other screening programmes, such as blood pressure measurement, are mentioned in the national plan [44].

The government’s new plan for CHW curriculum has a small focus on maternal health as stated above. However, it does not include the assessment or management of hypertensive disorders of pregnancy such as pre-eclampsia and eclampsia which are excluded from the training programme [43]. Furthermore, CHWs are not trained or provided with a blood pressure device to identify women with hypertension in the community [45]. Although CHWs are expected to recognise danger signs in pregnancy, including seizures and oedema, which can be related to hypertensive disorders of pregnancy, they are not trained nor equipped to confirm diagnosis or provide appropriate treatment [43, 45, 46].

## Discussion

In Mozambique, limited access to healthcare and skilled medical personnel contributes to high rates of maternal mortality. Many of these deaths would be preventable if adequate obstetric emergency care was available [47]. Systematic reviews examining CHW programmes worldwide have found that CHWs are effective in reducing maternal, neonatal and child mortality in resource-poor settings [48, 49], even when the focus of the programme is on prevention [50]. Mozambique has had a CHW programme since 1978, which did not include maternal health components. The programme survived several challenges and was revitalised in 2010. Despite the MDGs influence on the revitalization of the programme, contrary to what was observed regarding new approaches to the management of childhood illnesses at community level through the iCCM model [51], maternal health was introduced only with the focus on health prevention and promotion, and no effort to incorporate management of specific complications of pregnancy, such as pre-eclampsia and eclampsia, was made [52]. This can be in part explained by the fact that the WHO- and UNICEF-supported iCCM is purely centred on the management of the three deadliest illnesses in Sub-Saharan Africa (malaria, pneumonia and diarrhoea) for children under five [53]. However, more recently, there is increasing interest in expanding the iCCM of childhood illnesses to include other areas such as maternal health [54].

Despite the evidence that hypertensive disorders of pregnancy contribute significantly to maternal mortality in Mozambique [4–6] and that blood pressure assessment and management of hypertension can prevent morbidity and mortality due to pre-eclampsia [10], the absence of policy prevented the development of tools and trainings to appropriately equip CHWs to identify and manage these complications [45]. Unfortunately, even after the revitalization fostered by the MDGs, the revised CHW programme does not include pre-eclampsia and eclampsia-specific management [43].

It would be useful to evaluate whether training CHWs to provide diagnosis and management of pre-eclampsia and eclampsia with the aid of blood pressure devices, proteinuria tests, antihypertensive medications and magnesium sulphate could reduce maternal mortality and morbidity in settings where prompt access to health facilities is a challenge. These requirements for changes, however, may be met with significant opposition. For instance, there are laws preventing CHWs from prescribing certain medications [55]. Furthermore, concerns regarding the CHWs’ technical capacity to deal with more complex health conditions were identified as barriers in the implementation of iCCM of childhood illnesses in 2014 [51]. Finally, such tasks would have to be covered by adequate policies and guidelines. The Community Level Interventions for Pre-eclampsia (CLIP) Trial, which aims to reduce the weight of pre-eclampsia and eclampsia in maternal, perinatal and neonatal morbidity and mortality through the inclusion of screening and early management of severe pre-eclampsia by CHWs at the community, is currently underway in Maputo and Gaza provinces (NCT01911494) [56].

The revitalised CHW programme as a whole still faces numerous challenges due to significant discrepancies between policies and implementation. These include gaps in CHW coverage, as there are still not enough CHWs to meet the health needs of the population, and limited skills, training and supervision [16, 39]. Further, owing to the experienced difficulty in accessing health services, the community demands for curative and other services that are not formally recognised as part of CHWs’ tasks [27], inviting the enticement to perform some tasks illicitly.

There is uncertainty about the integration of CHWs into civil service and their long-term retention. In addition, reliance on NGOs and donor funding has led to an uneven distribution of CHWs in relation to the areas they serve as well as a disproportionate distribution of tasks to respond between preventive, curative, maternal and child health needs. Going forward, the dependence on external funding for the continuation of the CHW programme, when both external and government funding is declining, may hamper sustainability [16].

Although there seems to be a great deal of qualitative data supporting the potential role of CHWs in providing specific services other than health promotion [57], there is no quantitative data corroborating the effectiveness of CHWs in improving health outcomes in Mozambique. Further studies that assess the impact and sustainability of the revitalised programme are necessary before new plans to broaden its scope emerge.

## Conclusion

In Mozambique, where almost half of the population has no access to healthcare services, community health workers play a significant role in providing care to remote communities. Despite encouraging trends, maternal mortality remains high and the hypertensive disorders of pregnancy are one of the main contributors. Policies regarding the provision of prenatal care by CHWs are limited to health promotion and do not include the identification or emergency management of pregnancy complications, including pre-eclampsia. Studies to assess the impact of CHWs in providing maternal care to reduce maternal morbidity and mortality are recommended.

## Abbreviations

AIDS: Acquired immunodeficiency syndrome
AMOG: *Associação Moçambicana de Obstetras e Ginecologistas*
APE: *Agente polivalente elementar*
CHW: Community health worker
CISM: Centro de Investigação em Saúde de Manhiça
CLIP: Community Level Interventions for Pre-eclampsia
HIV: Human immunodeficiency virus
iCCM: Integrated Community Case Management
LMIC: Low- and middle-income countries
MDG: Millennium Development Goals
MoH: Ministry of health
NGO: Non-governmental organisation
UBC: University of British Columbia
UNAIDS: Joint United Nations Programme on HIV/AIDS
UNICEF: United Nations International Children’s Emergency Fund
WHO: World Health Organization
